# Harnessing Nth Root Gates for Energy Storage

**DOI:** 10.3390/e26110952

**Published:** 2024-11-06

**Authors:** Elliot John Fox, Marcela Herrera, Ferdinand Schmidt-Kaler, Irene D’Amico

**Affiliations:** 1 School of Physics, Engineering and Technology, University of York, York YO10 5DD, UK; elliot.fox@york.ac.uk; 2 Facultad de Ingeniería y Ciencias Básicas, Universidad Autónoma de Occidente, Cali 760030, Colombia; amherrera@uao.edu.co; 3 QUANTUM, Institut für Physik, Universität Mainz, D-55128 Mainz, Germany; fsk@uni-mainz.de

**Keywords:** quantum battery, quantum thermodynamics, ergotropy, quantum computation, quantum protocols

## Abstract

We explore the use of fractional controlled-not gates in quantum thermodynamics. The Nth-root gate allows for a paced application of two-qubit operations. We apply it in quantum thermodynamic protocols for charging a quantum battery. Circuits for three (and two) qubits are analysed by considering the generated ergotropy and other measures of performance. We also perform an optimisation of initial system parameters, e.g.,the initial quantum coherence of one of the qubits strongly affects the efficiency of protocols and the system’s performance as a battery. Finally, we briefly discuss the feasibility for an experimental realization.

## 1. Introduction

Advances in technology have enabled the construction and manipulation of microscopic systems, opening the door to develop experimental thermal machines which lie far from the thermodynamic limit [1,2,3,4,5,6,7,8]. Quantum effects and phenomena, such as the entanglement of particles, become extremely important in the description and modelling of these systems. These quantum phenomena can give advantages over classical systems in thermodynamic processes [8,9,10,11,12]. Quantum batteries represent an exciting application of quantum principles, with the potential to revolutionize energy storage for diverse scenarios. This kind of device has been implemented in multiple systems [13,14,15,16,17] and ergotropy has been the figure of merit used to quantify its effectiveness [18,19,20]. Quantum batteries may be based on collective quantum phenomena [13,21]. Hence, considering protocols using many-body interactions for these new technologies is of significant importance. Protocols inspired by quantum computing have been proposed for thermal machines, and the construction and testing of quantum many-body systems supporting the use of quantum logic gates is experimentally realisable using, among others, trapped ions [4,22], Rydberg atoms [23], and superconducting qubits [24]. While still in their early stages of development, theoretical and experimental studies have demonstrated the feasibility and potential benefits of quantum batteries [13,14,19,21,25,26,27]. Understanding their underlying principles and exploring practical implementations is the aim of this work. Replacing full Pauli gate operations with a fractional step-wise protocol has allowed for investigating the presence of quantum friction, i.e., the generation of quantum coherence in the instantaneous energy eigenbasis under a non-permuting protocol [28,29]. The experimental realization of this protocol has proven that quantum friction induces a violation of the work fluctuation dissipation relation, certifying an additional genuine quantum effect [30].

Here, we propose a fractional step-wise protocol with multiple qubits and using experimentally realisable Nth-root controlled-not logic gates (NRCGs) for charging and discharging a quantum battery. Repeat applications of NRCGs act as a trotterization of controlled-not logic gates; this has some analogy with collision models used in other works involving the charging of quantum batteries [22,31,32,33]. The NRCG stepped approach to the dynamics allows access to intermediate ergotropy states typically unobtainable using full controlled-not logic gates. This offers a greater control of the quantity of energy stored in the battery. NRCGs are feasible with current technology, and we find that, depending on the protocol and initial conditions, the use of an NRCG may provide overall more ergotropy than the use of its full controlled-not gate counterpart. We investigate the protocol for initial quantum coherences and find that this leads to improved performances as compared to a thermal state.

## 2. Theoretical Outline

We investigate various quantum thermodynamic protocols for charging a quantum battery. We express the scheme in a gate-based approach with quantum circuits of few-qubit systems, initialised and driven by a gate sequence, where we characterize the outcome of the protocol using the amount of ergotropy that has been generated.

### 2.1. System and Quantum Circuits

**Figure 1:**
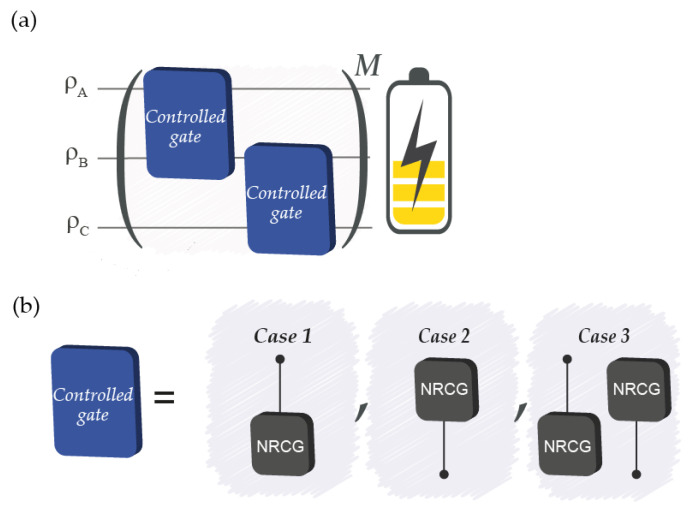
(**a**) Circuit diagram for the three-qubit protocols: the full protocol includes *M* iterations, each composed of two blocks of gates (blue squares). If M=N, the protocol is said to run over a “cycle”. (**b**) The protocols considered, i.e., case 1, case 2, and case 3, differ by the set of gates within each ‘controlled gate’ block, as indicated. A two-qubit protocol emerges from the circuit in panel (**a**) by removing qubit C and the second controlled gate block.

We consider a system composed either by two (A and B) or by three (A, B, and C) qubits that interact with each other through the use of NRCGs (Figure 1). These gates may generate entanglement between the component qubits. Qubits A and C are initialised each in a thermal state at temperatures $T_{A}$ and $T_{C}$, respectively, where $T_{A}>T_{C}>0$. The single-qubit thermal state is a Gibbs state defined as [34]

(1) $$\rho_{Gibbsj}\;=\;\left(1/Z_{j}\right)\exp^{-\beta_{j}H_{j}},$$

where $Z_{j}=\sum_{i}e^{-\beta_{j}\epsilon_{ij}}$ is the partition function, $\beta_{j}={\left(k_{B}T_{j}\right)}^{-1}$ is the inverse temperature parameter, $k_{B}$ is the Boltzmann constant, $\epsilon_{ij}$ are the eigenvalues of the single-qubit Hamiltonian. This is given by

(2) $$H_{j}\;=\;\left(\begin{array}{cc} \epsilon_{1j} & 0\\ 0 & \epsilon_{2j} \end{array}\right),$$

where j=A,B,C. As reference systems, we consider the ones with each qubit prepared in a thermal state: here, there are no initial quantum coherences. We compare these with the systems in which qubit B is prepared in a pure state; then, initial quantum coherence will carry through the qubits when a circuit is applied. Qubit B is initialised in a pure state as $\rho_{Pure}=\left|\psi \rangle \langle \psi \right|$, where $|\psi \rangle =\cos\left(\theta /2\right)|0\rangle +e^{i\phi }\sin\left(\theta /2\right)|1\rangle$ with a value of θ which ranges from 0toπ, while ϕ ranges from 0 to 2π. For this investigation, we will mainly focus on ϕ=0 and ϕ=π, though the full range of ϕ values will also be considered. Here, |0〉 is the ground state and |1〉 is the excited state. In this paper, energies are given in units of $\epsilon_{2B}$, which is then set to 1 in all calculations.

The initial Hamiltonian is non-interacting and of the form,

(3) $$H_{System}=\sum_{j}H_{j}$$

with $H_{j}$ given by Equation (2) and j=A,B or j=A,B,C for two and three qubits, respectively. We note that Equation (3) represents the Hamiltonian for the total system at any time, including at the point of measurement, except when NRCGs are applied, inducing interactions between qubits.

### 2.2. CNOT and Nth CNOT Root Logic Gates

The standard form of the CNOT gate for the two-qubit system is,

(4) $$CNOT_{A,B}\;=\;\left(\begin{array}{cccc} 1 & 0 & 0 & 0\\ 0 & 1 & 0 & 0\\ 0 & 0 & 0 & 1\\ 0 & 0 & 1 & 0 \end{array}\right),\;\;\;\;\;\;\;\;\;CNOT_{B,A}\;=\;\left(\begin{array}{cccc} 1 & 0 & 0 & 0\\ 0 & 0 & 0 & 1\\ 0 & 0 & 1 & 0\\ 0 & 1 & 0 & 0 \end{array}\right).$$

where the first subscript represents the control qubit and the second subscript, the target qubit. This type of gate may generate entanglement between two qubits, which adds a level of quantumness to the system. Entanglement of intermediate states, which a CNOT gate may achieve, is known to be necessary to gain a quantum advantage in battery charging operations [35]. The NRCG is a method of partially applying a CNOT gate; it is a unitary operation given by [36],

(5) $$\sqrt[{N}]{CNOT_{A,B}}\;=\;\left(\begin{array}{cccc} 1 & 0 & 0 & 0\\ 0 & 1 & 0 & 0\\ 0 & 0 & s & p\\ 0 & 0 & p & s \end{array}\right),\;\;\;\;\;\;\sqrt[{N}]{CNOT_{B,A}}\;=\;\left(\begin{array}{cccc} 1 & 0 & 0 & 0\\ 0 & s & 0 & p\\ 0 & 0 & 1 & 0\\ 0 & p & 0 & s \end{array}\right).$$

Here, $s=\frac{1}{2}+\frac{1}{2}e^{\frac{i\pi }{N}}$ and $p=\frac{1}{2}-\frac{1}{2}e^{\frac{i\pi }{N}}$.

A cycle is defined as N iterations of the basic circuit, corresponding to M=N in Figure 1a. In the limit of a large *N*, *N* consecutive applications of an NRCG with the same control and target qubits could be seen as a trotterization of the CNOT gate, aiming at explicitly implementing the gate as an adiabatic dynamic. In this sense, our protocols give explicit access to intermediate states, e.g., allowing for the opportunity to use states with different degrees of entanglement from the end result of the full CNOT gate.

### 2.3. Circuit

A schematic of the protocols applied is shown in Figure 1. We consider systems of two and three qubits and for each of them, we examine three ways of applying interactions, labeled 1, 2, and 3, see Figure 1b. Case 1 and 2 examine the dynamics when one of the qubits is never the control qubit, with this being either qubit A or C for the three-qubit systems. Case 3 examines the protocol where all qubits are either a control or target qubit over the course of one iteration. In all protocols, qubits A and C are always initialised in a thermal state using Equation (1), whereas qubit B is initialised in either a pure or a thermal state. This allows for probing how initial quantum coherences affect the system’s capabilities as a battery.

The system is evolved, corresponding to the quantum circuits in Figure 1 and to the evolution

(6) $$\rho_{sys}^{\left(f\right)}=U\rho_{sys}^{\left(0\right)}U^{†}$$

where $\rho_{sys}^{\left(0\right)}$ is the initial density matrix of the total system and $\rho_{sys}^{\left(f\right)}$ indicates the total density matrix after one iteration. The unitary *U* represents the controlled gates specified in Figure 1, as appropriate to each protocol, with NRCGs of the form in Equation (5).

We note that in all three-qubit protocols, as NRCGs are applied to different qubits or alternate different control/target qubits, successive iterations will not generate full CNOT gates. Instead, for Cases 1 and 2 and the two-qubit system, multiples of *N* iterations of the circuit will be equivalent to the application of multiple standard CNOT gates; however, fractions of *N* iterations will allow access to intermediate states in the evolution leading to a CNOT.

### 2.4. Ergotropy

Ergotropy is the maximum amount of work that can be extracted from a quantum system by means of a cyclic and unitary operation [37]. It is the primary measure used to explore the performance of a quantum battery [21]. For a state ρ of Hamiltonian *H*, the ergotropy is given by

(7) $$W_{max}=Tr\left[\rho H\right]-Tr\left[\sigma_{\rho }H\right],$$

where $\sigma_{\rho }$ is the passive state connected to ρ by a unitary transformation such that

(8) $$\sigma_{\rho }=\sum_{j}s_{j}|j\rangle \langle j|\;\mathrm{with}\;s_{j+1}\leq s_{j},$$

where $\left\{s_{j}\right\}$ are the eigenvalues of ρ and {|j〉} are the eigenstates of *H* [37]. No work can be extracted from a passive state, and for all unitaries *U*, $Tr\left(\sigma_{\rho }H\right)\leq Tr\left(U\sigma_{\rho }U^{†}H\right)$ [37,38].

## 3. Plan of the Paper and Anticipation of Main Results

The application of Nth-root gate operation between qubits unlocks a couple of assets, as we are able to follow the time evolution in a step-wise protocol and investigate the functioning of quantum battery protocols in more detail. Consequently, we consider three different protocols, highlighted in Figure 1 for varying numbers of iterations and different initializations. For a fair and meaningful comparison among all these systems and protocols, we will examine four measures of performance for an optimal battery:

- Ergotropy, which describes the maximum amount of work extractable at the end of the protocol;
- Ergotropy variation, which is the difference between the final and initial ergotropies, relevant when the initial state, like that here, is not a passive state;
- The ratio between ergotropy and the final energy of the system, which describes the fraction of energy that can be extracted as work;
- A figure of merit, which combines the ergotropy variation and the ratio.

We find that results are strongly affected by the initial coherence of qubit B, and the same circuit may lead to minimal or to maximal ergotropy, depending on the setting of the phase. Also, the overall largest ergotropy variation and figure of merit are obtained from a circuit with high initial coherence. For all protocols and all measures of performance, initializing qubit B in a pure state as opposed to a thermal state provides a substantial advantage.

## 4. Results

### 4.1. Ergotropy Scans for Two- and Three-Qubit Systems

The two-qubit system is initialised with qubit A thermalised in the Gibbs state Equation (1). We are using a reservoir where $k_{B}T_{A}=4\epsilon_{2B}$. For the three-qubit system, qubits A and C are initialised at $k_{B}T_{A}=4\epsilon_{2B}$ and $\;k_{B}T_{C}=0.4\epsilon_{2B}$, respectively. Qubit B is initialised in the pure state |ψ〉=cos(θ/2)|0〉+exp(iϕ)sin(θ/2)|1〉.

**Figure A1:**
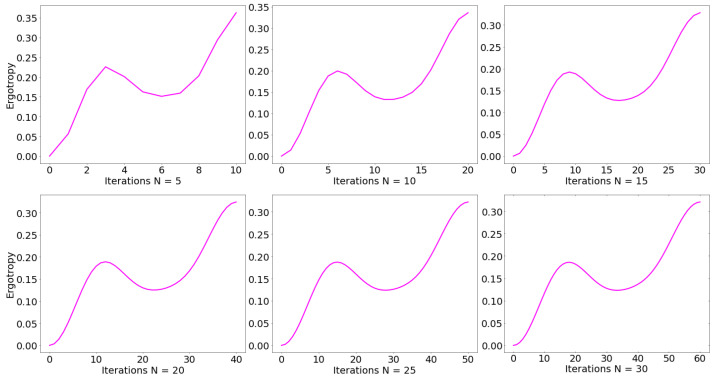
Ergotropy $W_{max}$ of the total system for 0≤ iterations ≤2N. For each panel, *N* is indicated on the *x*-axis; protocol corresponding to case 3 with three-qubit systems. Note that each panel corresponds to two cycles of iterations. Parameters: $\epsilon_{1j}=0,\;\epsilon_{2j}=1$ for j=A,B, and *C*.

One cycle is composed by *N* iterations. We choose N=15, with this value showing a smooth evolution of the state of the system as iterations are applied. Cycles with N>15 show no improvement but are increasing the run times of the simulations. Cycles with N<15 show increasing discontinuities, moving the evolution closer to the one using a complete CNOT gate (N=1). For details, see Appendix A, Figure A1.

**Figure 2:**
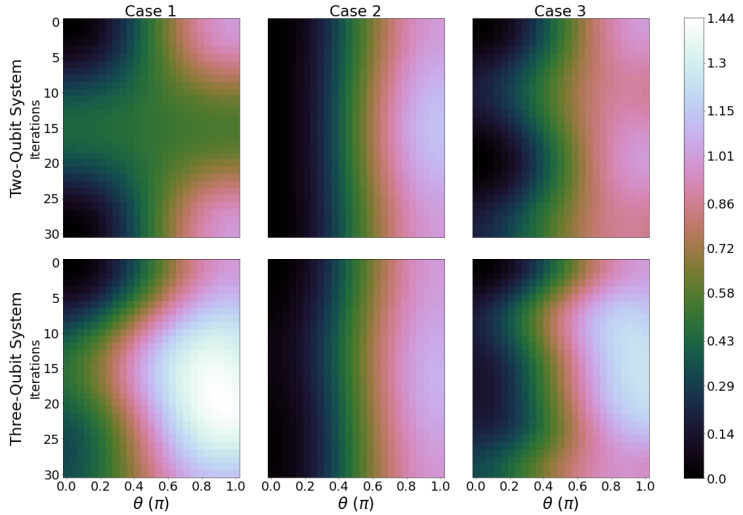
Ergotropy of the total system for 0≤θ≤π (*x*-axis) and 0≤ Iterations ≤30 (*y*-axis) for two-qubit systems (first row) and three-qubit systems (second row), where columns from left to right are case 1, 2, and 3, respectively. Brighter shades correspond to a greater value of ergotropy. Parameters are $\epsilon_{1j}=0\epsilon_{2B},\;\epsilon_{2j}=1\epsilon_{2B}$ for j=A,B, and *C*. $k_{B}T_{A}=4\epsilon_{2B}$ and $k_{B}T_{C}=0.4\epsilon_{2B}$. ϕ=π.

Each panel of Figure 2 shows results for the ergotropy calculated after 1, 2, ⋯,2N iterations, for 0≤θ≤π and ϕ=π. Increasing θ increases qubit B’s initial internal energy from $\epsilon_{1B}=0$ to $\epsilon_{2B}$. The first row refers to two-qubit systems, the second, to results for three-qubit systems, for all three cases described in Figure 1b, as labelled. We refer to each of the panels as an “ergotropy scan”.

The initial ergotropy (corresponding to zero iterations, top line of all panels) mainly depends on the initial energy of qubit B. The closer θ is to π, the larger the initial ergotropy of the total system. This can be seen across all cases for both two- and three-qubit systems. However, how the battery behaves for an increasing number of iterations depends on both the number of qubits and the circuit chosen, showing charging (ergotropy increasing with iterations) and discharging (ergotropy decreasing with iterations) regimes. For example, for case 1, θ=π and iterations increasing from 0 to 15, the two-qubit system would discharge while the three-qubit system would be charging.

Cases 1 and 3 show the largest difference in the ergotropy evolution, both with respect to the initial ergotropy value and when comparing the results for the two- and three-qubit systems. It is clear that in cases 1 and 3, there is an optimal number of iterations that would maximise the ergotropy, with the region of the largest ergotropy reached for three qubits, about 10 to 25 iterations and θ≳1.9 radians. For case 2, we find only a small variation in ergotropy as the system evolves.

**Figure A4:**
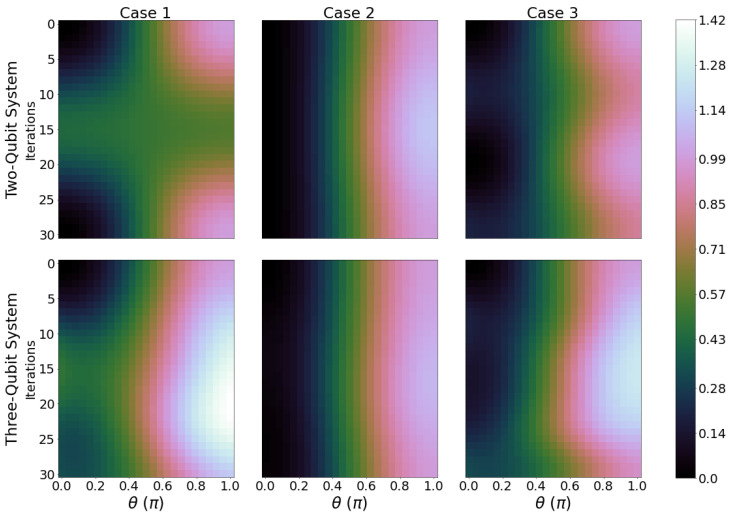
Ergotropy of the total system; parameters: $\epsilon_{1j}=0\epsilon_{2B},\;\epsilon_{2j}=1\epsilon_{2B}$ for j=A,B, and *C*. $k_{B}T_{A}=4\epsilon_{2B}$ and $k_{B}T_{C}=0.4\epsilon_{2B}$. ϕ=0. First row: Ergotropy of the total system for 0≤θ≤π (*x*-axis) and 0≤ iterations ≤30 (*y*-axis) for two-qubit systems where columns from left to right are cases 1, 2, and 3, respectively. Brighter shades correspond to a greater value of ergotropy. Second row: The same parameters as the first row but for three-qubit systems.

Results with ϕ=0 are similar (see Appendix C, Figure A4), with the three-qubit system for both case 1 and 3 showing the most notable variation in ergotropy, though their maximum ergotropy region gets shifted towards larger values of θ, θ≳2.5 radians, and they show a lesser variation in $W_{max}$ with iterations.

### 4.2. Comparison Between Circuits with Full CNOT and NRCGs

**Figure 3:**
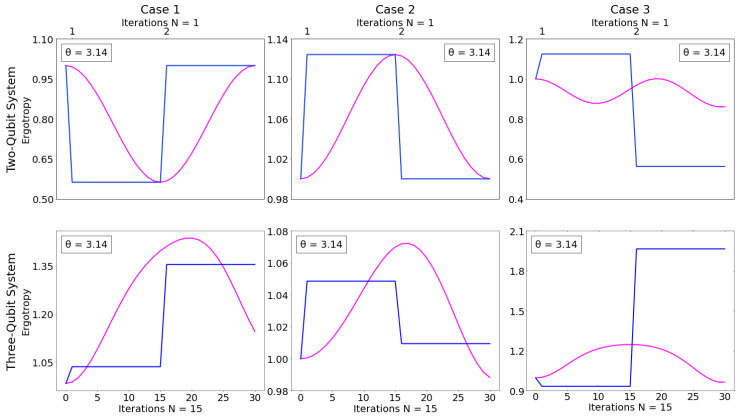
Comparing the ergotropy $W_{max}$ for the NCRG-protocol with 0≤ N ≤30 (magenta), and the standard CNOT (blue), in the case of the two-qubit systems (first row) and the three-qubit system (second row), where columns from left to right are cases 1, 2, and 3, respectively. The *y*-axis scale differs for each panel. The chosen value of θ is indicated in each panel. Parameters: Φ=π, $\epsilon_{1j}=0\epsilon_{2B},\;\epsilon_{2j}=1\epsilon_{2B}$ for j=A,B, and *C*; $k_{B}T_{A}=4\epsilon_{2B}$ and $k_{B}T_{C}=0.4\epsilon_{2B}$. Magenta line: N = 15; blue line: N = 1.

NCRGs can be interpreted as a stepped application of a full CNOT gate as explained more thoroughly in Section 2.2. We then wish to compare whether applying fractions of CNOTs instead of a full CNOT is advantageous in the present case. We compare the ergotropy over two cycles in which iterations have either of one CNOT or N = 15 steps of an NRCG, which is magenta. For each panel we consider θ such that the ergotropy of the system considered in the panel reaches its maximum when N = 15. The top left and top center panels in Figure 3, showing case 1 and 2, for the two-qubit systems operate with only a single gate per iteration. These particular systems demonstrate well how the NRCGs operate as a slower application of the full CNOT gate, with the systems’ ergotropy reaching the same values for both types of gates after one cycle. Circuits with the full CNOT gate allow us to reach only two specific values of ergotropy. However, NRCGs allow access to these and to all intermediate values. Such fine-tuning (increase and decrease) of ergotropy with the system’s evolution could prove advantageous when a particular value of ergotropy or the corresponding intermediate state are desired that are in-between the initial and final ergotropy and states generated by a full CNOT gate.

With the exception of cases 1 and 2 for two-qubit systems, NRCG-based cycles are not equivalent to cycles from corresponding circuits with CNOTs, even when considering the end results, see Figure 3. Apart from the easy reach of intermediate values of ergotropy, we can see two other ways in which the use of NRCGs could be advantageous: (i) at points where the ergotropy of the total system has a larger value than that of the standard CNOT gate within the same cycle; or (ii) when the maximum ergotropy reached using NRCGs is higher that the one of the corresponding CNOT gate circuits. The first possibility can be observed in all panels of Figure 3; the second can be seen in the three-qubit cases 1 and 2.

Similar results are found for the case of qubit B, inizialized in Φ=0. We note that a larger value of the ergotropy may be reached after the two cycles considered in Figure 3.

### 4.3. Ergotropy Variation for Two- and Three-Qubit Systems

Our systems do not start from a passive state, so the ergotropy $W_{max}$ at the end of the process may be influenced by the initial one. This is not a simple relation, as the systems studied undergo ‘discharging’ and ‘charging’ processes as the number of iterations increase, as discussed below.

In addition, when considering $W_{max}$ (Figure 2), a direct comparison between the two- and three-qubit systems is not easy to perform: when all common parameters are the same, the three-qubit system inherently starts from more energy and ergotropy than the two-qubit system by merit of having more qubits. For the dynamics considered, this almost always translates into acquiring more ergotropy by the end of the process (compare the first and second row in Figure 2). From this, we can argue that looking at the maximum ergotropy may not be a fair comparison between systems with different quantities of qubits. For these reasons, we introduce the ergotropy variation ($\Delta W_{max}$) which is defined by the following equation:

(9) $$\Delta W_{max}=W_{max}^{\left(f\right)}-W_{max}^{\left(0\right)},$$

where $W_{max}^{\left(f\right)}$ is the ergotropy of the final state and $W_{max}^{\left(0\right)}$ is the ergotropy of the initial state of the total system. Equation (9) allows the identification of two regimes with respect to the initial state, charging or discharging. A charging regime is characterised by a positive value of ergotropy variation and a discharging regime is characterised by a negative value of ergotropy variation. $\Delta W_{max}$ allows for a clearer comparison across different systems. For example, an equal or greater positive change in the two-qubit system compared to the corresponding three-qubit system may suggest that an easier-to-implement two-qubit circuit may be more suited to accrue energy as a battery.

**Figure 4:**
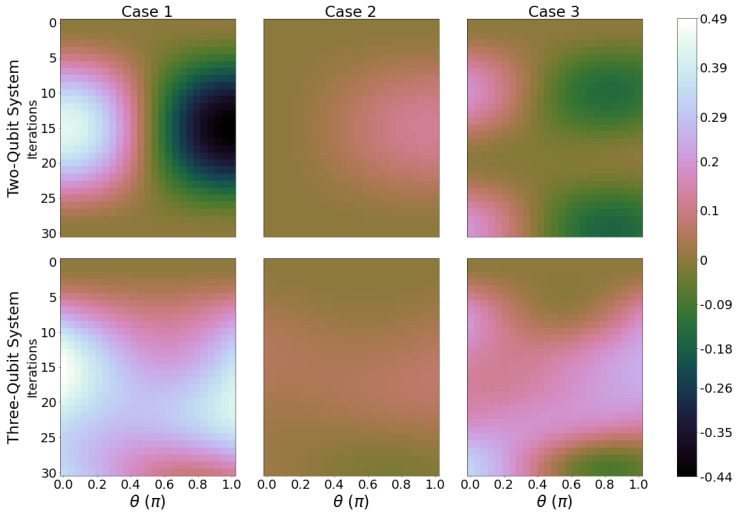
Ergotropy variation $\Delta W_{max}$ for ϕ=0. Otherwise, we have the same parameters as in Figure 2.

**Figure 5:**
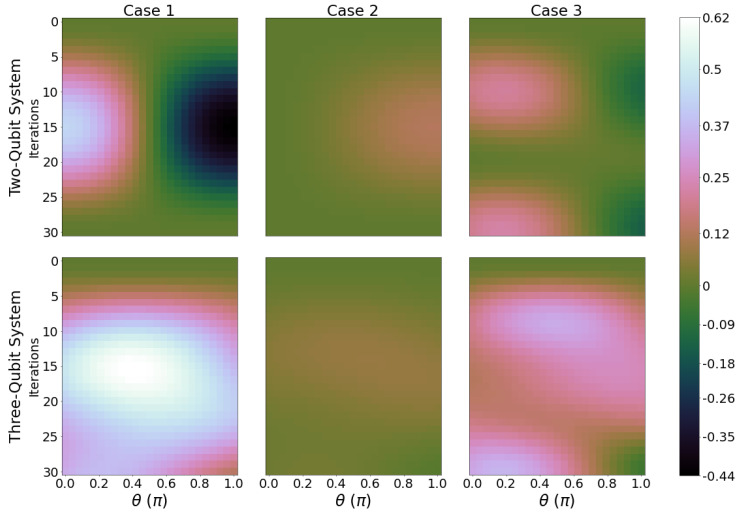
Ergotropy variation $\Delta W_{max}$ for ϕ=π, with the same parameters as in Figure 2.

$\Delta W_{max}$ is evaluated and plotted for ϕ=0, see Figure 4 and ϕ=π, see Figure 5. The corresponding panels for three-qubit systems show a dramatically different landscape, with the maxima no longer being constrained to θ≈π. We can see for case 2 that for the two- and three-qubit system, $\Delta W_{max}$ is small when compared to case 1 and 3, showing that it is not the optimal case for increasing or decreasing ergotropy from the initial state of the system. Cases 1 and 3 in Figure 4 display much larger peaks and troughs for $\Delta W_{max}$ with respect to case 2, indicating a stronger performance as a quantum battery with well-defined charging and discharging regimes. Comparison of cases 1 and 2, which correspond to inverted gates, indicates that having qubit A (thermalized initially at the higher temperature) be a control qubit produces more favourable changes in ergotropy. Also, the introduction of an extra two gates per iteration in case 3 fails to provide higher maxima in $\Delta W_{max}$ than case 1. Across all panels in Figure 4, the charging regions (positive $\Delta W_{max}$) are dominating over the discharging region, which is of advantage when looking to optimal conditions for operating a quantum battery. When qubit B is initialised with ϕ=π for three-qubit systems and cases 1 and 3, the maximum variation of ergotropy changes from 0.49$\epsilon_{2B}$ to 0.62$\epsilon_{2B}$, which is a 26.5% improvement in performance. For most cases, the maximum $\Delta W_{max}$ regions now lie closer to θ=π/2. This shows that the extra initial coherence enhances the charging performance in most circuits considered, and especially so for case 1 and 3 and three-qubit systems.

The ergotropy variation $\Delta W_{max}$ should not be the only quantity to consider when looking at the operating parameters for a quantum battery. A fair additional question could be how much of the final systems’ energy can be extracted as work. We will look at this, the ’ergotropy ratio’, in the next section.

### 4.4. Ergotropy Ratio for Two- and Three-Qubit Circuits

Here, we introduce the ergotropy ratio which is defined as,

(10) $$W_{ratio}=\frac{W_{max}}{Tr[\rho^{\left(f\right)}H]},$$

where $W_{max}$ is the ergotropy (Equation (7)) and $Tr[\rho^{\left(f\right)}H]$ is the internal energy of the system in its final state. The ergotropy ratio takes a value between 0 and 1, where a value closer to 1 signifies a larger quantity of extractable energy. The ergotropy ratio is a good measure of how efficiently a battery is performing [39]. With this in mind, here, $W_{ratio}$ is introduced to look for optimal initial conditions for an efficient quantum battery.

**Figure 6:**
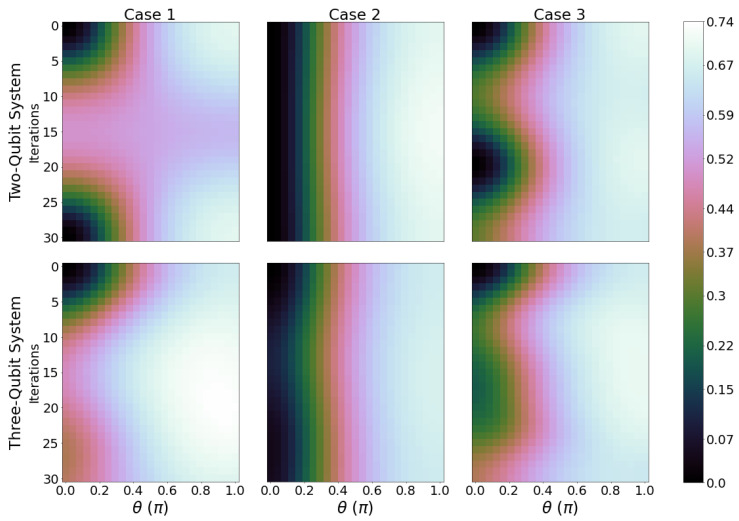
Ergotropy ratio $W_{ratio}$ for Φ=π, with the same parameters as in Figure 2.

**Figure A5:**
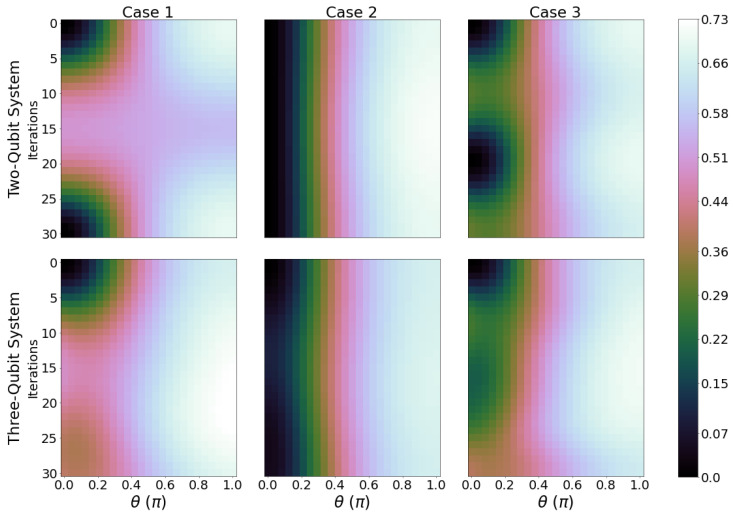
Ergotropy ratio $W_{ratio}$ for Φ=0, with the same parameters as Figure A4.

Figure 6 shows the ergotropy ratio for the total system for the same parameters as those in Figure 2, in particular, qubit B is initialised with Φ=π. For all cases, we obtain a greater value of ergotropy ratio when qubit B is initialised with a large value of θ, but not necessarily θ=π. The maximum values for $W_{ratio}$ across all panels in Figure 6 are similar, suggesting no particular advantage with using a three- instead of a two-qubit system. When initialising qubit B with Φ=π (see Figure 6), we observe a marked increased in areas of high $W_{ratio}$ for values of θ closer to π/2 for both two- (case 3) and three- (cases 1 and 3) qubit systems, supporting the advantage from a larger initial coherence. For comparison, results with qubit B initialised at Φ=0 are shown in Figure A5, Appendix C.

While $W_{ratio}$ is a good indicator for which initial conditions lead to a larger percentage of total energy that can be extracted as work, it does not show if that large percentage also corresponds to a region of high charging: indeed, $W_{ratio}$ and $\Delta W_{max}$ have quite different behaviours (e.g., compare Figure 4 and Figure A5). Ideally, one would like to optimize both quantities at the same time to quantitatively assess how well the initial conditions, system, and circuit considered perform as a battery. In the next section, we will introduce a related figure of merit.

### 4.5. Figure of Merit for Optimal Battery, Two- and Three-Qubit Systems

The figure of merit we propose to find the optimal circuit, number of iterations, and initial conditions for charging a quantum battery has the form

(11) $$FoM\left(\theta ,\Phi ,\mathrm{iter}\right)=\Delta W\times W_{ratio}.$$

This combination of quantities shows where the best balance of energy that can be extracted (ergotropy ratio) and the ergotropy increase from the initial condition (ergotropy difference) lie, giving a clearer indication of the optimal system.

**Figure 7:**
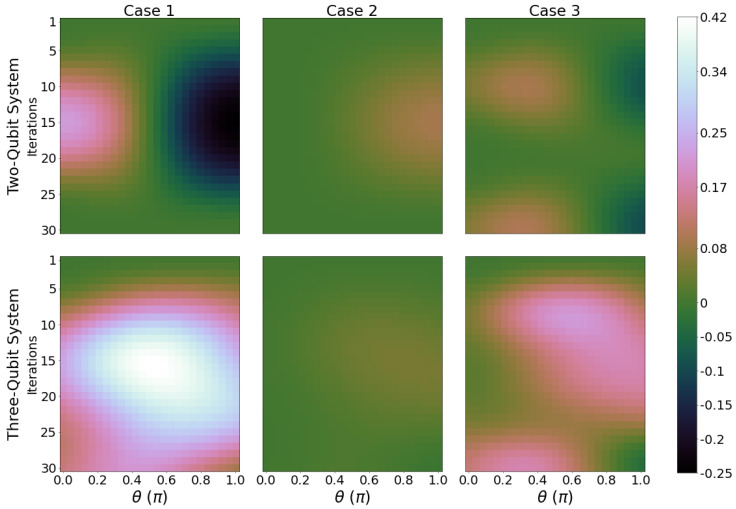
Figure of merit, FoM, with Φ=π, with the same parameters as Figure 2.

**Figure A6:**
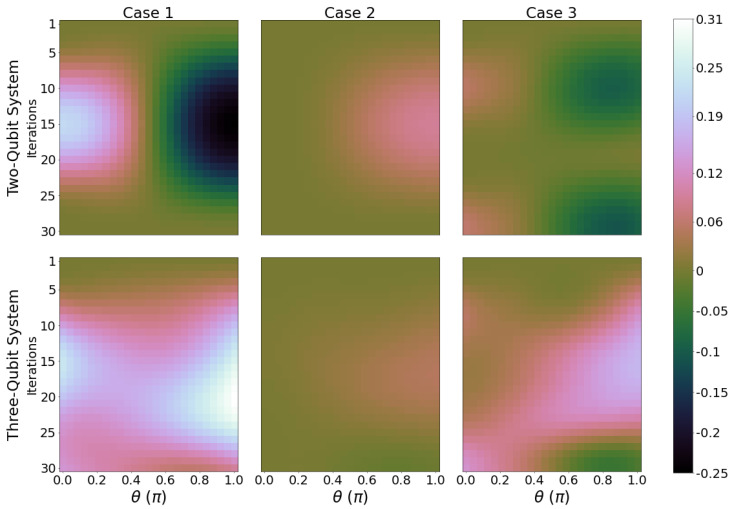
Figure of merit (FoM) for Φ=0, with the same parameters as Figure A4.

In Figure 7, we plot FoM for the same parameter scans of Figure 5 and Figure 6. In particular, qubit B is initialised with Φ=π. The results corresponding to Φ=0 are plotted in Appendix C, Figure A6. The topography of these graphs tends to mirror that of the variation in ergotropy, which is in general more variable with iterations than that of the ergotropy ratio. Interestingly, when Φ=0 (Figure A6), we see the highest FoM value regions when θ≈0 and θ≈π, suggesting that the system performs better as a battery when qubit B is initialised closer to a state with no coherences (Φ=0, with θ=0 or π). When comparing this to Figure 7 (Φ=π), the opposite is true, with the greatest value of FoM occurring when θ=π/2. The maximum value for the FoM is also 35% greater when initialising qubit B with ϕ=π with the system demonstrating a better performance when introducing initial coherences.

**Figure 8:**
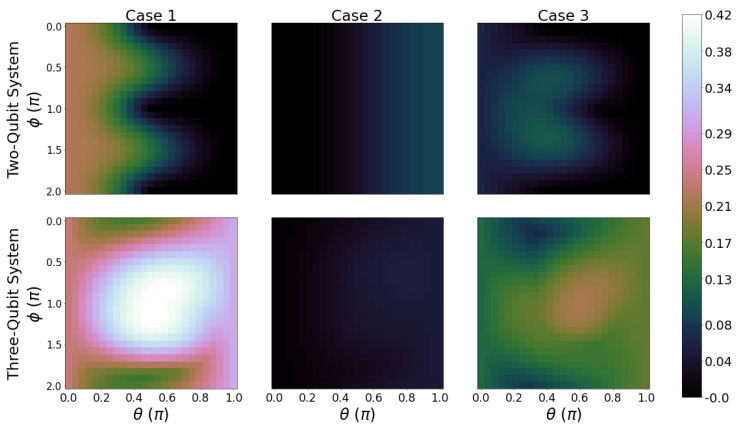
First row: Figure of merit of the total system in units of $\epsilon_{2B}$ for 0≤θ≤π (*x*-axis) and 0≤ϕ≤2π (*y*-axis) for two-qubit systems where columns from left to right are case 1, 2, and 3, respectively. Parameters: $\epsilon_{1j}=0\epsilon_{2B},\;\epsilon_{2j}=1\epsilon_{2B}$ for j=A,B, and *C*. $k_{B}T_{A}=4\epsilon_{2B}$ and $k_{B}T_{C}=0.4\epsilon_{2B}$. Brighter shades correspond to a greater value of the figure of merit. Second row: The same parameters as the first row but for three-qubit systems.

We now look at the dependence of FoM on both θ and ϕ, with 0≤ϕ<2π and 0≤θ<π. The values plotted in Figure 8 are the overall maximum FoM over 30 iterations for each given combination of θ and ϕ. Case 1 with the three-qubit system displays a clear advantage over the others (bottom left panel). Here, there is a large parameter region with a maximum value of FoM≈0.42, which is approximately double the next best cases (case 1 with two-qubits and case 3 with three-qubits) and which lies in the range of 0.63≲θ≲2.51 and 1.57≲ϕ≲4.71. When looking at the two-qubit systems for case 1 and case 3, we see that the value for the FoM has a double-peak pattern centered where the initial ϕ is either π/2 or 3π/4 and dropping to 0 for large values of θ. For two qubits, FoM acquires the highest values in case 1, for θ≈0, so with negligible initial coherence. For the three-qubit system, cases 3 and 1 have a similar trend, with the maximum value region around θ=π/2 and ϕ=π. This is a state with large initial coherence. Our results show for these cases a strong dependence of the FoM maximum value on ϕ, which is observed to be as important as θ when selecting optimal initial conditions. The least favourable circuit corresponds to case 2 (column 2): while it may present among the highest ergotropy ratios (Figure A5 and Figure 6), it shows an ergotropy variation close to zero (Figure 4 and Figure 5) which translates into a poor figure of merit. Interestingly, in contrast to the other cases, case 2 may perform better as a two-qubit system rather than a three-qubit system.

## 5. Comparison with Overall Thermal Initialization

In previous sections, we have seen that initial coherences tend to favor higher performances. Here, we compare the systems analysed so far with systems in which qubit B is initialised in a thermal state. Our results show a clear advantage of having qubit B initialised in a pure state.

**Figure 9:**
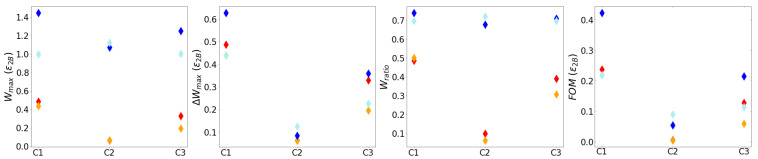
Comparison of performance measures: maximum ergotropy $W_{max}$ (first panel), maximum variation in ergotropy $\Delta W_{max}$ (second panel), maximum ergotropy ratio $W_{ratio}$ (third panel), and maximum figure of merit FoM (fourth panel) of the total system for case 1 (C1), case 2 (C2), and case 3 (C3), as labelled. Three-qubit system (blue diamonds), two-qubit system (light-blue), Three-qubit system with $B_{thermal}$ (red), two-qubit system with $B_{thermal}$ (orange). Parameters: $k_{B}T_{A}=4\epsilon_{2B}$ and $k_{B}T_{C}=0.4\epsilon_{2B}$. Maxima calculated over two cycles with N=15, 0≤ϕ≤2π and 0≤θ≤π.

In Figure 9, dark and light blue diamonds correspond to the original systems, i.e., with B initialised in a pure state, for three- and two-qubit systems, respectively, while red and orange diamonds map the systems with B initially being thermal ($B_{thermal}$ systems) for three- and two-qubit systems, respectively. The figure shows the results for the optimal initial configurations producing ergotropy (first panel), maximum ergotropy generation (second panel), the maximum ergotropy ratio (third panel), and maximum FoM (fourth panel) for case 1, case 2, and case 3, as labelled. Qubits *A* and *C* are always prepared in the thermal state Equation (1) with fixed temperatures $k_{B}T_{A}=4\epsilon_{2B}$ and $k_{B}T_{C}=0.4\epsilon_{2B}$ (only $k_{B}T_{A}$ applies when dealing with a two-qubit system). We note that each point in a panel may correspond to a different initial condition, depending on which initial value of θ and Φ will give the overall maximum of that particular quantity over 30 iterations.

To find the optimal values for the original systems, parameter scans for qubit B are performed for 0≤ϕ≤2π and 0≤θ≤π. For the initialisation of the $B_{thermal}$ systems, we scan the initial state of qubit B between the zero-temperature state (ground state) and the maximum energy and entropy state $\rho_{B}\;=\;\left(\begin{array}{cc} 1/2 & 0\\ 0 & 1/2 \end{array}\right)$. We vary the temperature in steps between the lowest and highest energy states, with the generic temperature defined by $k_{B}T_{B}=\langle E_{B}\rangle =Tr\left[\rho_{B}H_{0}\right]$.

The systematic advantage we observe in the original systems (blue and light blue diamonds) comes from the quantum coherences in the initial state of qubit B, as can be seen, e.g., from Figure 7 and Figure 8. This quantum advantage translates into a maximum ergotropy (first panel) about an order of magnitude higher for case 2, and two to five times higher for cases 1 and 3.

For the original systems, three-qubit set-ups outperform two-qubit ones for cases 1 and 3, while the opposite occurs for case 2. Case 1 for the original system with three qubits demonstrates the best performance across all measures.

## 6. Experimental Feasibility

The protocol requires several ingredients, each of which will be discussed separately: it is not challenging for any existing trapped ion quantum processor to operate a small set of 2 or 3 qubits. The initialization of qubit B into a superposition state is also a simple task for any quantum processor. The inizialization to Gibbs states of qubits A and C, however, is a non-standard operation, but it has been realized in some trapped ion quantum setups [4,30,40]. The step-wise execution of the gate, namely the Nth root two-qubit gate, requires the accurate calibration of the qubit diving, which is possibly a challenge when N is large. However, a step-wise execution appears to be feasible in trapped ion quantum processing, since recently it has been realized but for a single qubit operation [30] with N up to 10, and it is also well established for trotterized simulations like, for example, in the trapped ion quantum simulation of a high-energy system [41]. Finally, for the characterization of the quantum battery output, all methods for state tomography are readily available in trapped ions but also in any of the other platforms for quantum processors.

## 7. Discussion

A benefit of this current investigation is that NRCGs and the two–three qubit circuits we propose could be realised experimentally on a number of different platforms by using computational gates; this is an important next step in the development of quantum batteries, as noted by Campaioli et al. [20]. When looking at other investigations that inspired this work, the focus is generally on maximum work extraction, which we first looked at in Section 4.1. The importance of initial coherences for the ergotropy was noted Refs. [42,43]. While we find that the ergotropy is positively influenced by initial coherence, the other measures of performance we consider are even more affected, as summarized below. The power of charging, and by extension the efficiency [13,19,44,45], are also used in quantum battery research. The power is investigated in Appendix B.

Our investigation compares a set of three different protocols with two or three identical qubits as working fluid. One of the qubits is initialised in a pure state. The idea to establish which protocol and system is best as a battery under which circumstances has led us to consider a set of measures of performance. Hence, we focus not only on how much work or power can be extracted, but also on which fraction of the system’s internal energy is extractable and characterised this by the ergotropy ratio (Equation (10)), used also in [39]. We find that regions with better performance depend on the initialisation of the system just as much as the method used for charging, generally favouring initial qubit B states where θ>π/2 and hence with some degree of population inversion. However, comparison with results from corresponding initial states where the off-diagonal elements of qubit B are set to 0 demonstrates that initial coherences significantly enhance the ergotropy ratio for θ<π/2.

The difference between initial and final ergotropies ΔW is introduced as a performance indicator. When looking at optimising initial conditions for battery charging, this is arguably a metric that is more important than just the total ergotropy, especially when initialising the system in a state other than the ground state. With this, we can discriminate clear winners among the cases implemented, with case 2 under-performing against cases 1 and 3. This indicator also clarifies charging and discharging regimes, with positive regions corresponding to charging and negative to discharging. We find that initial coherence in qubit B is definitely beneficial to charging, improving the maximum ergotropy difference by 26.5% (compare results for ϕ=0 and ϕ=π). Results for the power in Appendix B confirm the importance of initial coherences for enhanced performances.

With the aim of identifying systems that would be good overall performers, we then introduce a figure of merit, FoM, combining the ergotropy ratio and the variation in ergotropy. By scanning all initial conditions, the FoM allows us to identify the protocol of case 1 with three-qubits as the overall best for use as a battery. The FoM of this system has a marked dependence on the initial condition, favouring initialisations with the largest initial coherence in qubit B, providing us with a clear quantum advantage.

In the last part of this paper, we compare performances for all protocols with corresponding systems with qubit B initialised in a thermal state. The main takeaway from these comparisons is that introducing a pure-state initial component is advantageous across the board, and for all measures of performance, see Figure 9. Initial coherences lead to overall maxima for some measures of performance.

Further investigations could expand this work by exploring the effect of intermediate measurements on the protocols’ efficiency [46] and utilizing alternative Nth root computational gates and different quantum thermal machines. We found that, depending on the protocol, the best performer could be either the two- or the three-qubit system: future research could explore the scaling (and its consistency) for increasing the number of qubits. Another avenue of future investigation could be the use of a collective bath [47] and of global collective charging [48] to verify if, in our case, they would improve performances.

A challenge that this system and others could face experimentally is the loss of coherences, whether from dissipation or through direct measurement [46]. How ergotropy and related measures of performance would be affected would depend on the dissipation mechanisms specific to the hardware. However, as hardware for quantum computers has already been optimized to perform a very large number of gates within the relevant decoherence times, quantum batteries based on quantum circuits should also share this advantage.

## 8. Conclusions

In this paper we have shown that circuits containing Nth-root CNOT gates could be advantageously used as quantum batteries. We evaluate the circuits’ performance using five different measures—ergotropy, power, ergotropy difference and ratio, and a figure of merit—and considering two- and three-qubit circuits. Our results show consistently that having one of the qubits initialised in a pure state is highly advantageous with respect to using a thermal state with the same energy: high performances are strongly and positively influenced by initial quantum coherences. For any given initial condition, and at difference with standard CNOT gates, iterating circuits with Nth-root CNOT gates allows access to a fine distribution of values of ergotropy: this flexibility could be advantageous for tailoring the same battery to different working needs. The systems and protocol we propose are experimentally feasible with the current technology.

## Figures and Tables

**Figure A2:**
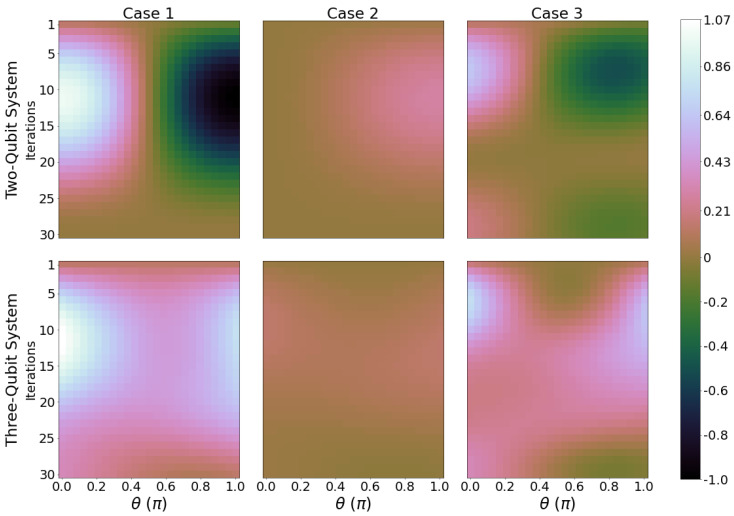
Power ($P_{\Delta W_{max}}$) of the total system for 0≤θ≤π (*x*-axis) and 0≤ iterations ≤30 (*y*-axis); First row: Two-qubit systems where columns from left to right are cases 1, 2, and 3, respectively. Brighter shades correspond to a greater value of power. Parameters: $\epsilon_{1j}=0\epsilon_{2B},\;\epsilon_{2j}=1\epsilon_{2B}$ for j=A,B, and *C*. $k_{B}T_{A}=4\epsilon_{2B}$ and $k_{B}T_{C}=0.4\epsilon_{2B}$. ϕ=0. Second row: The same parameters as the first row but for three-qubit systems.

**Figure A3:**
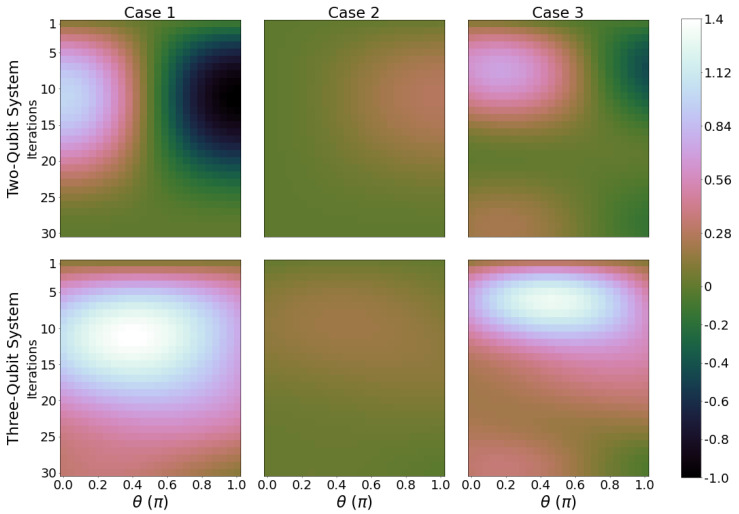
The same as Figure A2, but for ϕ=π.

## Data Availability

The original contributions presented in the study are included in the article, further inquiries can be directed to the corresponding author.

## Appendix A

Figure A1 shows how changing *N* affects the ergotropy for case 3 and the three-qubit system. We observe a “smoothing” of the evolution of the ergotropy for increasing *N*.

## Appendix B

Adapting from Refs. [13,19], the power of charging for our protocols up to the α iteration is

(A1) $$P_{\alpha }=\frac{\langle W_{\alpha }\rangle }{T_{\alpha }},$$

where $T_{\alpha }=\alpha /M$ is the process duration up to the α iteration and *M* is the total number of iterations considered (here, M=30 corresponding to two cycles). For α=M, $T_{\alpha }=1$. $\langle W_{\alpha }\rangle$ is the average work defined as $\langle W_{\alpha }\rangle =E^{\alpha }-E^{i}$, where $E^{\alpha }=Tr\left[\rho^{\alpha }H\right]$.

When evaluating the power of a quantum battery, there is more depth than just the difference in the average internal energy of the system. While the average internal energy plays a role, this is not a one-to-one comparison with the ergotropy, which as thoroughly discussed in the main article, being a better measure for quantum battery performance. With that in mind, we introduce,

(A2) $$P_{\Delta W_{max}}=\frac{\Delta W_{max}}{T_{\alpha }},$$

where $P_{\Delta W_{max}}$ is the power of charging or discharging and $\Delta W_{max}$ is the variation in ergotropy up to the α iteration. Equation (A2) characterises the rate at which the system generates ergotropy. The total, ergotropy is not used as we are interested in charging from some initial state ($\rho^{i}$) that has a non-zero ergotropy.

Figure A2 and Figure A3 follow a somewhat similar trend to that of Figure 4 and Figure 5, respectively (where Figure 4 and Figure 5 show $\Delta W_{max}$). Indeed, for the three-qubit systems, the maximum values of $P_{\Delta W_{max}}$ are reached when ϕ=π (Figure 5) and areas with θ≈π/2, where initial coherence is large. When ϕ=0 (Figure 5), the regions of high power are achieved when θ≈0orπ, for which we have lower initial coherence. These maxima appear for similar values as the maxima of $\Delta W_{max}$, but at earlier iterations, see Figure 4 and Figure 5. Looking at Equation (A1), we see that as iterations increase, a reduction in power is expected, which is what we see in Figure A2 and Figure A3. This is why we see the power maxima at lower iterations than that of the maxima in Figure 4 and Figure 5. Directly comparing the maxima for Figure A2 and Figure A3, we see a larger maximum power reached when ϕ=π with a value of 1.42$\epsilon_{2B}$ compared with 1.07$\epsilon_{2B}$, giving a 32.7% increase.

## Appendix C

For completeness, in this Appendix, we present results for parameter scans with qubit B initialised with Φ=0 and for the following quantities: The ergotropy of the total system (Figure A4), the ergotropy ratio $W_{ratio}$ (Figure A5), and the figure of merit FoM (Figure A6).
